# A combination of CMC and α-MSH inhibited ROS activated NLRP3 inflammasome in hyperosmolarity stressed HCECs and scopolamine-induced dry eye rats

**DOI:** 10.1038/s41598-020-80849-2

**Published:** 2021-01-13

**Authors:** Ying Lv, Chenchen Chu, Ke Liu, Yusha Ru, Yan Zhang, Xiaoxiao Lu, Yichen Gao, Caijie Zhang, Shaozhen Zhao

**Affiliations:** 1 Tianjin Medical University Eye Hospital, Eye Institute and School of Optometry, Tianjin International Joint Research and Development Centre of Ophthalmology and Vision Science, Tianjin, 300384 China; 2 Tianjin Medical University Eye Hospital, Tianjin Medical University Eye Institute, College of Optometry and Ophthalmology, Tianjin Medical University, Tianjin, China

**Keywords:** Eye diseases, Corneal diseases

## Abstract

An important mechanism involved in dry eye (DE) is the association between tear hyperosmolarity and inflammation severity. Inflammation in DE might be mediated by the NLRP3 inflammasome, which activated by exposure to reactive oxygen species (ROS). A combination of carboxymethylcellulose (CMC) and α-melanocyte stimulating hormone (α-MSH) may influence DE through this mechanism, thus avoiding defects of signal drug. In this study, we assessed whether treatment comprising CMC combined with α-MSH could ameliorate ocular surface function; we found that it promoted tear secretion, reduced the density of fluorescein sodium staining, enhanced the number of conjunctival goblet cells, and reduced the number of corneal apoptotic cells. Investigation of the underlying mechanism suggested that the synergistic effect of combined treatment alleviated DE inflammation through reduction of ROS level and inhibition of the NLRP3 inflammasome in human corneal epithelial cells. These findings indicate that combined CMC + α-MSH treatment could ameliorate lesions and restore ocular surface function in patients with DE through reduction of ROS level and inhibition of NLRP3 signalling.

## Introduction

The prevalence of dry eye (DE) is reportedly between 5 and 34% worldwide; it is higher among women and elderly individuals^1^. The definition of DE was revised during the Dry Eye Workshop II; the current definition comprises an imbalance in tear film homeostasis and onset of concurrent ocular symptoms, including tear instability, hyperosmolarity, inflammation, and neurosensory abnormalities^2^. DE symptoms such as foreign body sensation, burning, and stinging can impact productivity and reduce quality of life in affected individuals; if not treated in a timely manner through appropriate therapeutic methods, the chronic inflammatory state involved in DE may cause vision loss or blindness.

Recommended treatment of DE comprises graded treatment in accordance with disease severity. Therapeutic agents include artificial tears, anti-inflammatory drugs, immunosuppressive drugs, and autologous serum. However, artificial tears only provide temporary alleviation of symptoms and are ineffective in patients with severe disease. Long-term usage of glucocorticoids can cause intraocular hypertension, while immunosuppressants are expensive and may aggravate symptoms in patients who receive them as treatment; moreover, the preparation of autologous serum is complex and difficult to commercialise. Finally, surgical approaches are required for treatment of patients with severe disease^3,4^. Thus, there is an urgent need for an effective drug that can be used in clinical practice for treatment of DE.

α-Melanocyte stimulating hormone (α-MSH) is an immunoregulatory neuropeptide derived from melanin precursors; it is mainly produced by the pituitary gland, hypothalamus, and various peripheral tissue cells. Notably, it was the first immunoregulatory neuropeptide identified in the eyes^5^. α-MSH contributes to numerous key processes in the eyes, including growth and development, anti-inflammatory function, and immune regulation^6–9^. Our previous studies revealed that α-MSH could alleviate DE symptoms in rats by repairing corneal epithelial damage^10^; moreover, it can inhibit overexpression of pro-inflammatory factors on the ocular surface by activation of the PKA–CREB and MEK–ERK pathways^11^. Carboxymethylcellulose (CMC) is a high-viscosity polymer that has been used in artificial tear treatment for nearly 70 years. It has strong adhesion capability that enables prolong retention of tears on the ocular surface^12–14^. However, for patients with DE who exhibit corneal epithelium damage, simple tear supplements are insufficient treatment. Robust therapeutic gels to promote the repair process are needed, combined with non-steroidal drugs or low-concentration glucocorticoids^15^. However, patient compliance is often poor due to the frequency of medication and use of multiple agents.

Here, we hypothesised that CMC combined with α-MSH exhibits a synergistic effect in treatment of DE. To test our hypothesis, we measured tear secretion, corneal fluorescein sodium staining, and the numbers of conjunctival goblet and corneal apoptotic cells. To investigate the underlying mechanisms of the combined effects, we also performed in vitro examination of reactive oxygen species (ROS) level, as well as the levels of NLRP3 inflammasome and caspase-1 expression.

## Results

### CMC + α-MSH ameliorated corneal dysfunction in rats with scopolamine-induced DE

**Figure 1:**
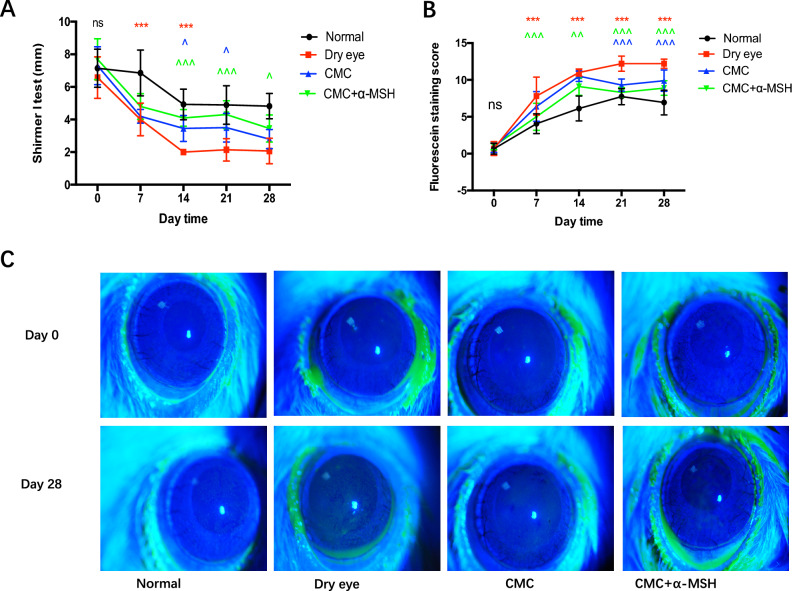
Combined CMC + α-MSH treatment ameliorated ocular surface dysfunction in rats with scopolamine-induced dry eye. Rats were divided into normal (black line), dry eye (red line), CMC (blue line), and α-MSH (green line) groups. Schirmer’s test (**A**) and corneal fluorescein staining (**B**) were performed weekly. Representative images of corneal fluorescein staining before and after the experiment are shown (**C**). Data are shown as mean ± SEM, n = 10 per group. *p < 0.05; **p < 0.01; ***p < 0.001.

**Figure 2:**
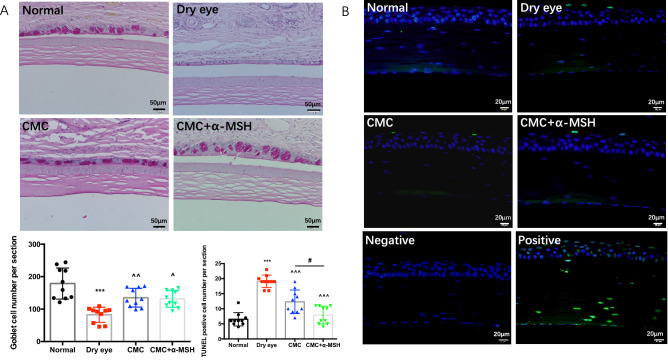
Combined CMC + α-MSH treatment enhanced the number and size of conjunctival goblet cells and reduced the number of apoptotic cells in cornea. Rats were divided into normal, dry eye, CMC, and CMC + α-MSH groups. The number and size of conjunctival goblet cells were examined by PAS staining. Representative images of PAS staining are shown. The number of conjunctival goblet cells was quantified (**A**). The number of apoptotic cells was examined by TUNEL staining. Representative images of TUNEL staining are shown. The number of apoptotic cells was quantified (**B**). Data are shown as mean ± SEM, n = 10 per group. *, comparison of dry eye group and normal group; ^, comparison of CMC treatment group or combined CMC + α-MSH treatment group and hypertonic group; #, comparison of CMC treatment group and combined CMC + α-MSH treatment group; ns, not significant. *, ^, # *p* < 0.05; **, ^^^^*p* < 0.01; ***, ^^^^^*p* < 0.001.

As shown in Fig. 1, the Shirmer I test (SIt) value was considerably higher in the CMC + α-MSH group than in the NaCl group at 7, 14, and 21 days after intervention (all *P* < 0.01). Corneal fluorescence staining scores were significantly lower in the CMC, α-MSH, and CMC + α-MSH groups than in the NaCl group (all *P* < 0.05); the lowest score was observed in the CMC + α-MSH group. No significant differences were found between the DE and NaCl groups in terms of SIt and corneal fluorescence staining scores at various time points (all *P* > 0.05). Figure 2A shows that the numbers of goblet cells were much higher in the CMC, α-MSH, and CMC + α-MSH groups than in the DE and NaCl groups (all *P* < 0.01). The CMC + α-MSH group also exhibited fewer apoptotic corneal epithelial cells, compared with the DE group, as shown in Fig. 2B.

### CMC + α-MSH treatment reduced NLRP3 and caspase-1 protein expression levels in rats with scopolamine-induced DE

**Figure 3:**
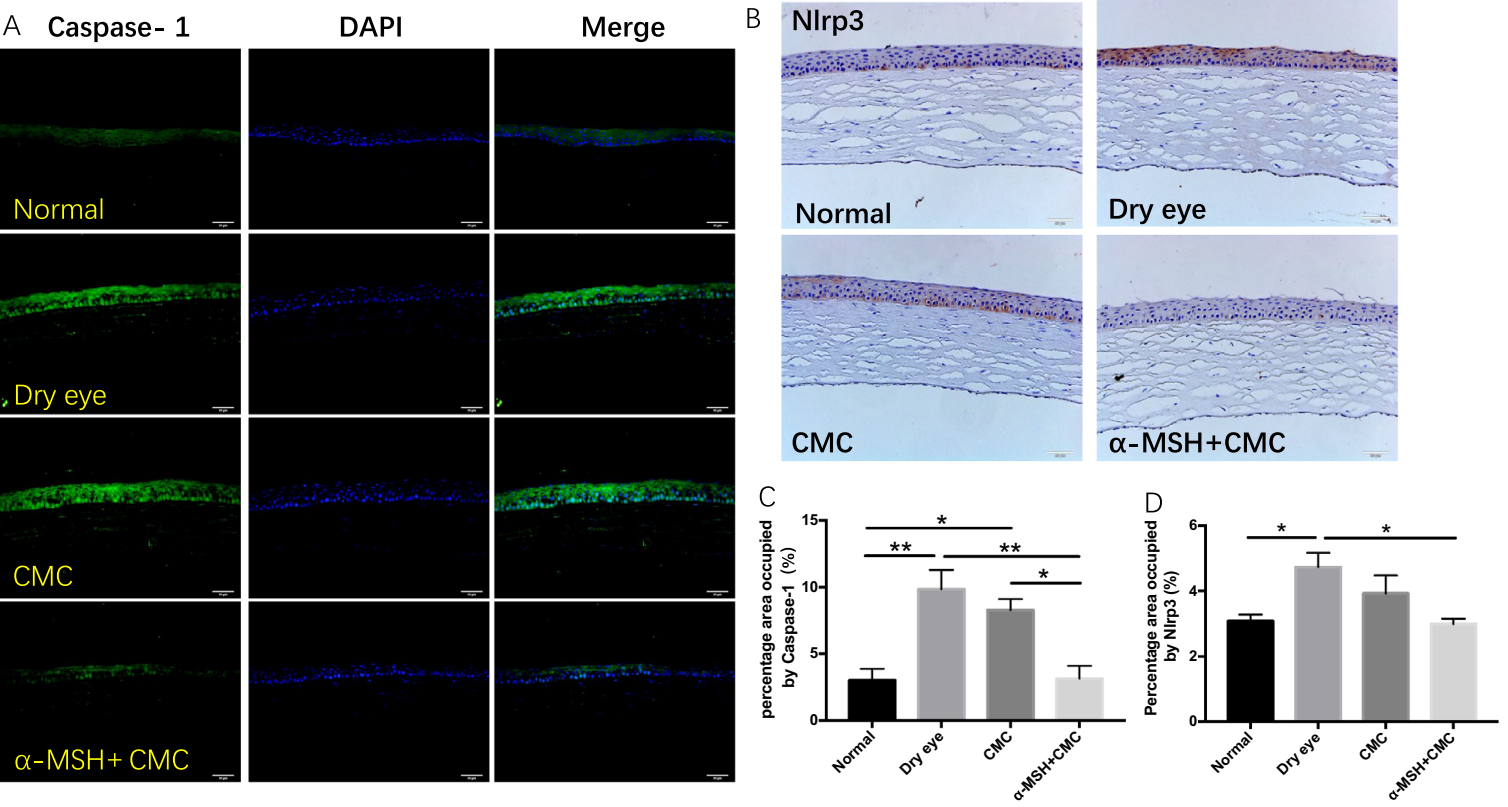
Combined CMC + α-MSH treatment reduced Nlrp3 and Caspase-1 expression levels in rats with scopolamine-induced dry eye. Rats were divided into normal, dry eye, CMC, and CMC + α-MSH groups. The Caspase-1 expression was examined by immunofluorescence staining. Representative images of immunofluorescence staining are shown (**A**). The expression level was quantified (n = 5, **C**). The Nlrp3 expression was examined by immunohistochemistry. Representative images of immunohistochemistry are shown (**B**) The expression level was quantified (n = 6, **D**). Data are shown as mean ± SEM. * *p* < 0.05; ** *p* < 0.01; *** *p* < 0.001.

Immunohistochemistry and immunofluorescence analyses were performed to verify the protein expression levels of NLRP3 and caspase-1. Figure 3 showes that NLRP3 and caspase-1 protein expression levels were much higher in the DE group than in the normal group (*p* < 0.01). Combined CMC + α-MSH treatment was able to reduce these levels. However, protein expression levels in the group that received CMC treatment alone were nearly identical to levels in the DE group.

### CMC + α-MSH enhanced the viability of stressed human corneal epithelial cells (HCECs) in a hyperosmotic environment

**Figure 4:**
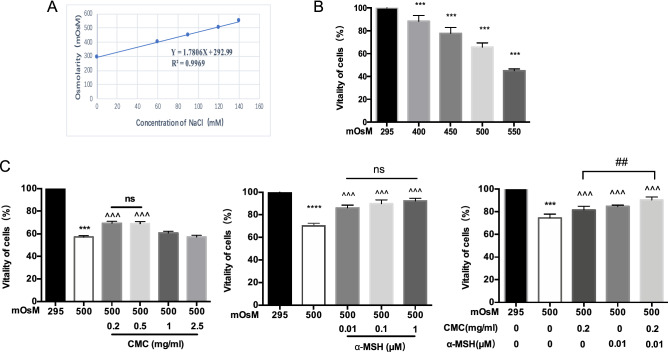
Combined CMC + α-MSH treatment enhanced the viability of HCECs exposed to hyperosmotic environmental stress. Linear relationship between concentrations of sodium dissolved in DMEM/F12 medium and corresponding osmotic pressure (**A**). Three samples were taken from different cell sites during osmotic pressure determination. HCEC viability was measured by CCK assay after treatment with several concentrations of sodium-containing medium, with and without CMC and α-MSH. Absorbance values represent relative cell viability (n = 6, **B**,**C**). Three independent experiments were performed. Data are shown as mean ± SEM. *, comparison of hypertonic group and normal group; ^, comparison of CMC treatment group or α-MSH treatment group and hypertonic group; ns, not significant. *, ^^^*p* < 0.05; **, ^^^^*p* < 0.01; ***, ^^^^^*p* < 0.001; ns, not significant.

Figure 4A shows a linear relationship between NaCl concentration and osmolarity. Notably, HCEC viability decreased with increasing osmotic pressure, as shown in Fig. 4B. After incubation in a 550-mOsM environment for 24 h, the number of adherent HCECs was significantly reduced, compared with incubation in a 295-mOsM environment. HCECs did not exhibit significant morphological changes after 24 h of incubation in 400-, 450-, and 500-mOsM environments. Cell viability was measured as an indicator of the effective concentrations of CMC and α-MSH. Figure 4C shows that 0.2 mg/mL and 0.5 mg/mL CMC tended to reduce HCEC viability in a hyperosmotic environment, compared to untreated cells, but these reductions were not statistically significant; notably, 1 mg/mL and 2.5 mg/mL CMC did not influence cell viability. Thus, 0.2 mg/mL CMC was chosen for further experiments. Furthermore, α-MSH tended to exhibit a concentration-dependent effect on HCEC viability under hyperosmotic stress; however, these results did not significantly differ among the three groups (concentrations of 0.01, 0.1, and 1 μM α-MSH). Therefore, 0.01 μM α-MSH was chosen for further experiments. The efficacy of combined treatment with 0.2 mg/mL CMC and 0.01 μM α-MSH was greater than that of either drug alone.

### CMC + α-MSH promoted the migration and tight junction integrity of HCECs exposed to hyperosmotic environmental stress

**Figure 5:**
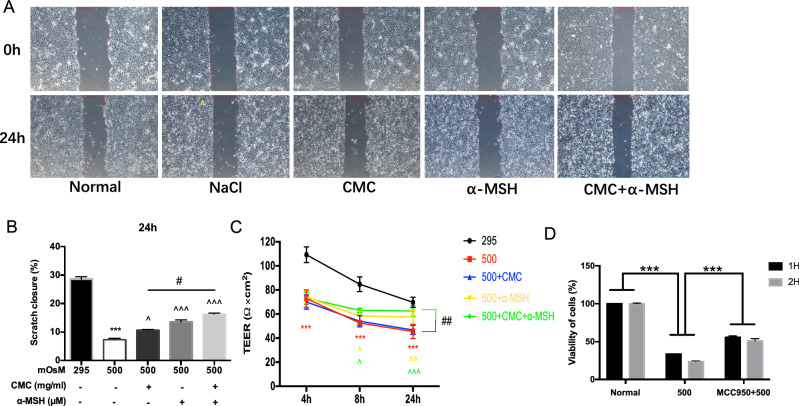
Combined CMC + α-MSH treatment promoted migration and tight junction formation by HCECs exposed to hyperosmotic environmental stress. Representative images immediately after and at 24 h after scratch formation are shown (**A**). The migration ratio was quantified (**B**). The resistance value between upper and lower chambers represented the tight junction ability of HCECs (**C**). The HCEC viability was measured by CCK assay after treatment in sodium medium and MCC950. Three independent experiments were performed (**D**). Data are shown as mean ± SEM, n = 6 per group.

Combined treatment with CMC and α-MSH has been shown to reduce the fluorescein sodium staining score in a rat model of DE (Fig. 1B), indicating that the combined use of CMC and α-MSH could promote the repair of rat corneal epithelium in vivo. To investigate whether treatment with CMC, α-MSH, or CMC + α-MSH could promote the repair of HCECs in vitro, cell migration ability was assessed by means of scratch tests. As shown in Fig. 5A,B), a 500-mOsM environment reduced HCEC migration ability. CMC, α-MSH, and CMC + α-MSH promoted the migration of HCECs in a hyperosmotic environment; combined treatment with CMC and α-MSH resulted in scratch closure that did not significantly differ from closure in the 295-mOsM environment (Fig. 5A,B).

Preliminary results showed that HCECs exhibited peak transepithelial electrical resistance values between the fifth and seventh days, while the values began to decrease after the seventh day; accordingly, starvation treatment was performed on the sixth day. As shown in Fig. 5C, exposure to a 500-mOsM hyperosmotic environment reduced the strength of intercellular tight junctions and weakened the corneal epithelium barrier function; conversely, α-MSH and CMC + α-MSH treatments promoted the ability of HCECs to form tight junctions after 8 h of hyperosmotic stimulation, whereas CMC treatment did not. After 24 h, HCEC barrier function in the CMC + α-MSH group was restored to a level similar to that of the normal group (Fig. 5C). To demonstrate that NLRP3 plays an important role in the pathogenesis of DE, NLRP3 inhibitors (MCC950) was used to determine the cells activity. Figure 5D demonstrates that cell viability was reduced in cells grown in a hyperosmotic environment, compared with cells in the normal group (*p* < 0.01). Treatment with MCC950 resulted in recovery of cell viability (*p* < 0.01).

### Combined CMC + α-MSH treatment reduced the ROS level, IL-1*β *in supernatant and mRNA levels of *NLRP3* and *IL-1β* in HCECs exposed to hyperosmotic environmental stress

**Figure 6:**
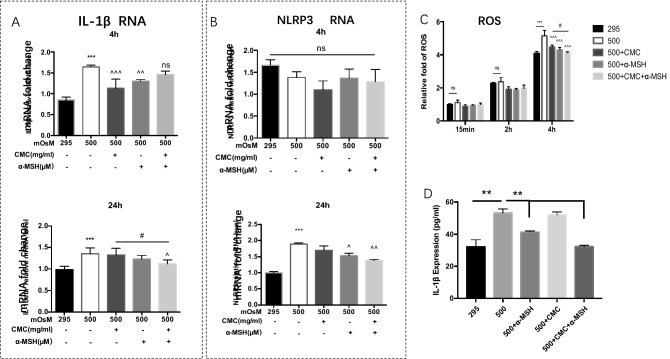
Combined CMC + α-MSH treatment reduced mRNA levels of NLRP3 and IL-1β in HCECs exposed to hyperosmotic environmental stress. mRNA levels of NLRP3 and IL-1β among groups after 4 h (**A**) and 24 h (**B**) of treatment are shown. Relative expression levels were analysed using the comparative threshold cycle (2^−∆∆Ct^) method and normalised to GAPDH gene expression. ROS levels were compared among groups treated with CMC, α-MSH, and CMC + α-MSH in a hyperosmotic environment, or left untreated (**C**). IL-1β expression in cell supernatant (**D**). Data are shown as mean ± SEM. Three independent experiments were performed, n = 6 per group. *, comparison of dry eye group and normal group; ^, comparison of α-MSH treatment group or combined CMC + α-MSH treatment group and hypertonic group; #, comparison of CMC treatment group and combined CMC + α-MSH treatment group; ns, not significant. *, ^#^*p* < 0.05; **p < 0.01; ***p < 0.001.

As shown in Fig. 6A,B, the mRNA levels of *NLRP3* did not significantly differ among groups after 4 h; however, the *IL-1β* mRNA level was upregulated in the group exposed to a hyperosmotic environment alone, compared with the normal group (P < 0.001). However, mRNA levels of both *NLRP3* and *IL-1β* were significantly upregulated after 24 h in the group exposed to a hyperosmotic environment alone, compared with the normal group (both P < 0.001). CMC treatment alone did not produce a significant difference in the mRNA levels of *NLRP3* and *IL-1β*, compared with the group exposed to a hyperosmotic environment alone; conversely, α-MSH treatment alone led to downregulation of the *NLRP3* mRNA level (P < 0.05), but did not influence the transcription of *IL-1β*. Notably, the group that received combined treatment exhibited reduced mRNA levels of both *NLRP3* and *IL-1β* (both P < 0.05). The ROS level in HCECs began to increase after 15 min of growth in a hyperosmotic environment; after 4 h, it was significantly higher than the level observed in the normal group. The reduction in ROS level in the CMC + α-MSH group was more noticeable than the reduction in either group that received single drug treatment (Fig. 6C). Analysis with enzyme-linked immunosorbent assays (ELISAs) revealed that IL-1β protein expression level in supernatant increased in the group exposed to a hyperosmotic environment alone; this elevated level was reduced by α-MSH and combined CMC + α-MSH treatments. Furthermore, the IL-1β protein expression level tended to be lower in the group that received combined CMC + α-MSH treatment, although this difference was not statistically significant. Notably, treatment with CMC alone did not cause a significant difference in IL-1β protein expression level, compared with the group exposed to a hyperosmotic environment alone (Fig. 6D).

### Combined CMC + α-MSH treatment reduced NLRP3 protein expression in HCECs exposed to hyperosmotic environmental stress

**Figure 7:**
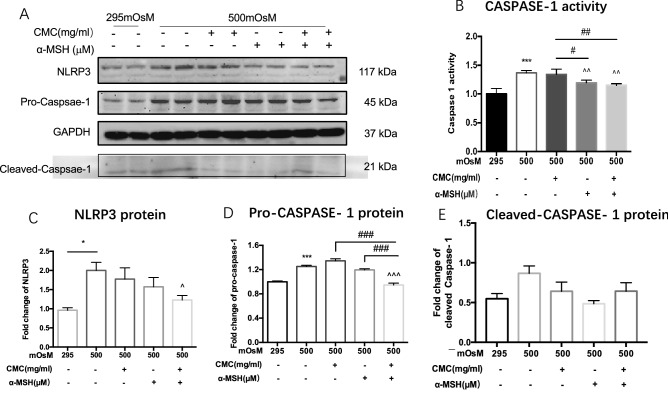
Combined CMC + α-MSH treatment reduced the ROS level and NLRP3 expression in HCECs exposed to hyperosmotic environmental stress. (**A**–**C**) Western blot analysis of cell lysates derived from HCECs among the indicated groups (n = 6). (**D**,**E**) Caspase-1 activities were compared among groups (n = 6). Data are shown as mean ± SEM. Three independent experiments were performed. The samples derive from the same experiment and that gels/blots were processed in parallel. (**A**) Showed the cropped gels and blots and full-length blots are presented in Supplementary Fig. S7A. *, ^#^*p* < 0.05; **, ^##^*p* < 0.01; ***, ^###^*p* < 0.001.

After 24 h of hyperosmotic stimulation, the protein expression levels of NLRP3 and pro-caspase-1 were significantly elevated, compared with levels in the normal group (all P < 0.05). The protein expression levels of pro-caspase-1 in the CMC and α-MSH groups did not significantly differ from the level observed in the group exposed to a hyperosmotic environment alone (both P > 0.05), while the protein expression levels of NLRP3 and pro-caspase-1 were significantly reduced in the CMC + α-MSH group (both P < 0.05, Fig. 7A,C,D). The cleaved- caspase- 1 expression showed the same trend as pro-caspase-1 without statistical difference (Fig. 7E). Compared with the normal group, caspase-1 activity was elevated in the group exposed to a hyperosmotic environment alone (P < 0.001); the activity was not influenced by CMC alone, but was reduced upon α-MSH treatment or combined CMC + α-MSH treatment (both P < 0.01, Fig. 7B).

### Combined CMC + α-MSH treatment reduced the enhancement of NLRP3 expression and caspase-1 activity in lipopolysaccharide (LPS)-stimulated HCECs exposed to hyperosmotic environmental stress

**Figure 8:**
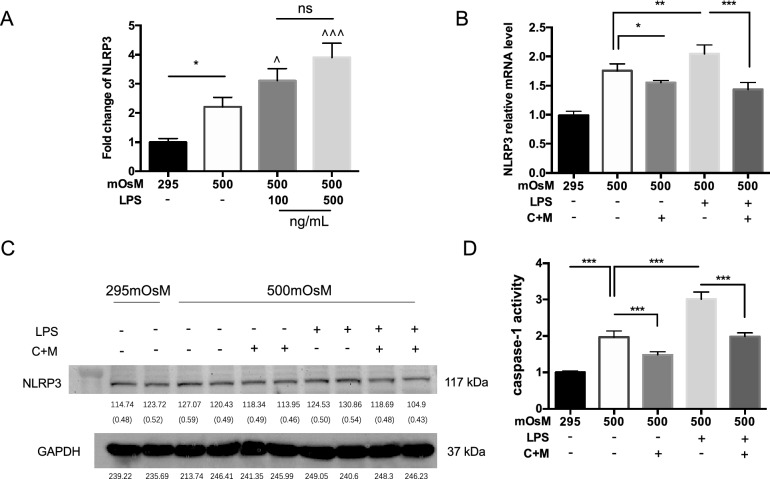
Combined CMC + α-MSH treatment reduced the enhancement of NLRP3 expression and caspase-1 activity in LPS-stimulated HCECs exposed to hyperosmotic environmental stress. (**A**) mRNA transcription of cell lysates derived from HCECs treated with LPS (100 ng/ml, 500 ng/ml) in a hyperosmotic environment or left untreated for the indicated times (n = 6). (**B**,**C**) Western blot and real-time PCR analyses of NLRP3 expression in HCECs treated with or without LPS (100 ng/ml), with or without CMC + α-MSH, in a hyperosmotic environment or left untreated for the indicated times (n = 6). (**D**) Examination of caspase-1 activity among the groups indicated in (**B**) (n = 6). Data are shown as mean ± SEM. Three independent experiments were performed. The samples derive from the same experiment and that gels/blots were processed in parallel. (**C**) Showed the cropped gels and blots and full-length blots are presented in Supplementary Fig. S8C. *p < 0.05; **p < 0.01; ***p < 0.001.

Stimulation with 100 ng/mL and 500 ng/mL LPS led to significant enhancement of the *NLRP3* mRNA expression level in corneal epithelium, compared with its level in the group exposed to a hyperosmotic environment alone (P < 0.05); there was no significant difference in *NLRP3* mRNA expression level between the two LPS treatments (Fig. 8A). Therefore, 100 ng/mL was chosen as the final concentration for further experiments. Following LPS stimulation of HCECs in a hyperosmotic environment, combined CMC + α-MSH treatment significantly inhibited NLRP3 expression in mRNA (P < 0.05, Fig. 8B). This trend was also reflected in protein level (Fig. 8C). As shown in Fig. 8D, caspase-1 activity in the group exposed to LPS stimulation alone was significantly enhanced, compared with its activity in the group exposed to a hyperosmotic environment alone (P < 0.001); this enhancement was inhibited by combined CMC + α-MSH treatment.

## Discussion

Inflammation and tear hyperosmolarity have recently been included as characteristics of DE syndrome^16^. Tears that exhibit a hyperosmotic nature can cause indirect or direct ocular surface damage through a series of inflammatory reactions. This hyperosmotic environment causes inflammation of the cornea and apoptosis of both conjunctival epithelial cells and goblet cells^17,18^; these changes cause tear film instability and onset of DE. Therefore, various anti-inflammatory agents have been investigated for treatment of DE. In the present study, we confirmed that combined CMC and α-MSH treatment exhibited a synergistic effect in a rat model of scopolamine-induced DE, as well as HCECs exposed to hyperosmotic environmental stress. Inflammasomes are key molecular regulators that play important roles in inflammation. The effects of inflammasomes in DE have been established^19^; their inhibition generally aids in reduction of inflammation. Inflammasome inhibition also helps to reduce the inflammation associated with DE. Calcitriol has been shown to significantly inhibit the expression of NLRP3 inflammasome-related genes and IL-1β production in cells exposed to hyperosmotic environmental stress. Notably, calcitriol protects cells from hyperosmotic injury by inhibiting the expression of the ROS–NLRP3–IL-1β axis^20^.

Consistent with the prior findings, our results in vivo showed that Nlrp3 and Caspase- 1 level in protein significantly increased in cornea of rat with DE. Furthermore, our results in vitro also showed that ROS level and *IL-1β* mRNA transcription both significantly increased after 4 h of hyperosmotic stimulation, whereas the *NLRP3* mRNA level did not change. After 24 h of hyperosmotic stimulation, the mRNA levels of *NLRP3* and *IL-1β* were considerably increased. However, Chen et al. reported that ROS levels significantly increased after 15 min of exposure to a 500-mOsM environment, while mRNA levels of both *NLRP3* and *IL-1β* increased within 4 h^21^. Although the timing differed between our study and the study by Chen et al., changes in ROS occurred before changes in *NLRP3* mRNA transcription, which indicated that activation of the NLRP3 inflammasome was affected by ROS due to hyperosmotic stress. Notably, considerable accumulation of ROS is needed to cause downstream changes. The discrepancy between experiments (i.e., 4 h compared with 15 min) may be related to differences in reagents and methods.

LPS is an activator of NLRP3 that has been investigated in previous studies; notably, adenosine triphosphate (ATP) is needed to mediate the effects of LPS on NLRP3 activation. However, upon exposure to LPS in a hyperosmotic environment, the mRNA and protein levels of NLRP3 are elevated. To explain this, Gyzman et al. investigated ATP levels in human corneal and conjunctival epithelial cells exposed to a hyperosmotic environment, as well as in patients with DE; they found that ATP levels were significantly higher following hyperosmotic stress, compared with levels in cells and patients in normal groups^22^. Therefore, in a hyperosmotic environment, ROS presumably activates NLRP3. Furthermore, NLRP3 activation is enhanced by LPS stimulation, thereby increasing the mRNA and protein levels of NLRP3. Yang et al. found that upon activation of cells by LPS, caspase-11 induced opening of the cell membrane channel pannexin-1 and two subsequent effluxes of K + to activate NLRP3; it also released ATP as a purinergic receptor (P2X7R) agonist and promoted further activation of NLRP3^23^. Therefore, following enhanced activation of NLRP3 by LPS in a hyperosmotic environment, combined CMC + α-MSH treatment can inhibit the LPS-mediated induction of elevated NLRP3 mRNA and protein levels, but this inhibition is limited.

Recently, there have been increasing studies have been carried out for the combination of drugs. For example, Chen et al. mixed CMC with hyaluronic acid (HA) in 2015 to form a new type of artificial tear for animal experiments; they investigated whether this treatment produced a better therapeutic effect on DE induced by an intelligent controlled environment system^14^. In the same year, Simmons et al. studied the effects of CMC + HA eye drops on alleviation of DE symptoms and signs in a multicentre clinical trial^24^. In 2017, Labetoulle and colleagues combined CMC, HA, and osmoprotectants in clinical trials; they reported that the combination was noninferior to HA alone^25^. However, the effects of different compositions of artificial tears are limited. Our previous study revealed that α-MSH was an effective anti-inflammatory and anti-apoptotic agent that may be useful in DE treatment^11^. In the present study, we investigated whether the combination of α-MSH with an artificial tear agent was more effective, using both in vitro and in vivo experiments. As shown in Fig. 1A, tear secretion was much greater in the combined CMC + α-MSH treatment group than in the CMC group, indicating that combination treatment provided a greater effect. This therapeutic advantage was also demonstrated in subsequent in vitro experiments including assessments of cell viability and migration, as well as inhibition of inflammasome expression. When we began this investigation, we selected cell viability to screen our osmotic pressure model. Notably, we found that the osmolarity selected for assessment of cell viability in our experiment was similar to the level used in previous studies. In the relevant literature, the osmolarity is often 450 mOsM or 500 mOsM; however, the corresponding sodium concentration was not consistent between our study and the prior studies. In our study, a freezing point osmometer was used and corrected before each measurement; each concentration was measured at least three times. Thus, differences between our findings and the results in prior studies may be related to differences in osmotic pressure instruments.

In conclusion, our findings indicate that combined CMC + α-MSH treatment is superior to treatment with CMC alone in a rat model of scopolamine-induced DE syndrome. We also found that combined CMC + α-MSH treatment exerted a protective effect via inhibition of NLRP3 and reduction of ROS level. The effective treatment and explicit mechanism suggest a combined preparation of CMC and α-MSH would be a novel eye drop treatment for patients with DE. In vivo investigations of the underlying mechanism of this combined treatment and its other effects on the ocular surface will be the focus of our future studies.

## Methods

### Animals

Forty female Wistar rats (6–8 weeks of age, 160–180 g) were purchased from the Chinese Academy of Military Medical Sciences (Beijing, China). The animals were housed at 25 °C ± 1 °C with 40% ± 5% humidity under 12-h light–dark illumination cycles. The animals were provided food and water ad libitum. The rats were randomly divided into normal, DE, CMC, and CMC + α-MSH groups. The DE, CMC, and CMC + α-MSH groups were injected subcutaneously with 6 mg/mL scopolamine hydrobromide, four times per day for 28 days. This approach has been demonstrated to successfully induce DE in rats^11,26^. Rats in the CMC group received topical treatment with 0.5% CMC (Allergan, Dublin, Ireland) eye drops, twice per day; rats in the CMC + α-MSH group received 1 × 10^−3^ mg/mL α-MSH (Millipore US) dissolved in 0.5% CMC eye drops, twice per day. The treatment regimen was initiated on the first day of the modelling protocol. The SIt and corneal fluorescence staining were performed at 7-day intervals. On the 28th day, eyeballs were collected from all rats. These experimental procedures were approved by the Experimental Animal Ethic Committee of Tianjin Medical University (SYXK 2009-0001) and adhered to the tenets of the Association for Research in Vision and Ophthalmology and the Ophthalmology Statement for the Use of Animals in Ophthalmic and Vision Research.

### Shirmer I test

A phenol red cotton thread was placed in the lower fornix for 30 s at one-third of the distance from the lateral canthus, without the use of anaesthesia. The red portion of the thread was measured and recorded in mm.

### Corneal fluorescein staining

Ten microliters of 0.1% sodium fluorescein were dripped into each rat’s inferior conjunctival sac. Three artificial blinks were performed and fluorescein staining of the corneal surface was observed under a slit-lamp microscope with cobalt blue light. The cornea was divided into four quadrants and each quadrant was scored as follows, according to the staining degree and area of fluorescein: 0, no staining; 1, scattered punctate staining (fewer than 30 punctae); 2, punctate staining (more than 30 punctae), but not dispersive; 3, serious dispersive staining, but no plaque formation; 4: plaque staining. The sum of the four quadrants was recorded as the corneal fluorescein staining score.

### Tissue staining

Staining (both haematoxylin and eosin and period acid–Schiff [PAS]) was performed as previously described^11^. Fixed intact eyeballs were dehydrated in an ethanol gradient, cleared in xylene, embedded in paraffin, and sectioned (5 μm thickness) along the sagittal plane. Corneal tissue morphology was observed by haematoxylin and eosin staining and assessed using an optical microscope; conjunctival goblet cell morphology was observed by PAS staining (as described below) and assessed using an optical microscope (Olympus Optical Co. Ltd., Tokyo, Japan). Ten to 15 paraffin sections at matching positions of the anterior segment were stained with a PAS Kit (Sigma-Aldrich, St. Louis, MO, USA) and counterstained with haematoxylin.

### TUNEL assay

Terminal deoxynucleotidyl transferase dUTP nick end labelling (TUNEL) staining was performed as previously described^11^. Briefly, sections of each eyeball were subjected to TUNEL fluorescein staining using an In Situ Cell Death Detection Kit (Roche Diagnostics, Branford, CT, USA); two sections served as respective positive and negative controls. After they had been stained, the slides were mounted with 6-diamidino-2-phenylindole (DAPI) reagent (Life Technologies, Grand Island, NY, USA). Images were acquired using uniform optical parameters by the cellSens Standard electronic system (Olympus Optical Co. Ltd.) under a fluorescence microscope (BX51, Olympus Optical Co. Ltd.)^11^.

### Immunohistochemistry

Immunohistochemistry was conducted to verify the expression of NLRP3 at protein levels. In brief, eyeballs (n = 3 per group) were fixed in 10% natural formalin, paraffin-embedded, and sagittally sectioned (5 μm thickness). Tissue sections were incubated with rabbit anti-rat NLRP3 antibody (1:50, Abcam, UK) at 4 °C overnight. Sections were then washed and incubated with horseradish peroxidase-conjugated goat anti-rabbit secondary antibody (Abcam, UK) for 2 h at room temperature. Sections were counterstained with haematoxylin and observed under a BX51 microscope (Olympus Optical Co. Ltd.). Images were acquired using the cellSens Standard electronic system (Olympus Optical Co. Ltd.) with uniform optical parameters. The intensity of 3,3′-diaminobenzidine immunostaining was quantified using ImageJ software^27^.

### Immunofluorescence staining

Freshly excised eye-balls were snap frozen in Tissue-Tek optimum cutting temperature compound (Sakura Finetechnical, Tokyo, Japan); frozen sections of 6-μm thickness were fixed in 4% paraformaldehyde for 15 min, permeabilised with 0.3% Triton X-100 for 5 min, and blocked with goat serum (zhongshan, Beijing, China) for 1 h. Tissue was incubated with primary antibody (anti-caspase-1; 1:500 dilution; Novus, UK) at 4 °C overnight. Tissue was then washed three times with phosphate-buffered saline (PBS) and then incubated with secondary antibody (green, Abcam, UK) for 1 h (1:500). For detection of cell nuclei, DAPI was added to the mounting medium. Tissues were imaged via fluorescence microscopy.

### Cell culture and groups

The human corneal epithelial cell line was donated by Professor Zuguo Liu of the Institute of Ophthalmology of Xiamen University in 2015. Cells were cultured in basal medium (DMEM/F12) mixed with 6% fetal bovine serum (Gibco, CA, USA), 1% penicillin (100 U/ml, Gibco), 1% streptomycin (100 μg/ml, Gibco), 0.1% recombinant human epithelial growth factor (7 ng/ml, Sigma) and 0.35% fetal bovine insulin (7 g/ml, Sigma); growth medium was changed at 2-day intervals. Cells were passaged when they reached 80% to 90% confluence; cells from the third to fifth passages that exhibited good morphology were used for experiments. On the day after cells had been seeded, they were washed twice with Dulbecco’s PBS (Gibco). Cells were then starved with fetal bovine serum-free basal medium overnight (16 h); they were then treated with hyperosmotic medium alone, or with hyperosmotic medium combined with CMC, α-MSH, or CMC + α-MSH. Cells were pre-incubated with CMC, α-MSH, and CMC + α-MSH at twofold greater than the desired final concentration for 30 min before hyperosmotic stimulation; an equal volume of hyperosmotic medium with twofold greater than the desired final concentration was then added to reach the desired concentration. Two LPS (Sigma, US) concentrations of 500 ng/ml and 100 ng/ml were used in this study. Pre-incubation with CMC + α-MSH was performed for 30 min, as above; an equal volume of hyperosmotic medium containing LPS and twofold greater than the desired final concentration of CMC + α-MSH was added. Mcc950 used the same incubation method as LPS. Cells in all groups were then incubated for 24 h.

### CCK-8 assay

Several concentrations of DMEM/F12 medium dissolving sodium were measured by freezing point osmotic measurement. Cells were seeded in 96-well plates at a density of 1 × 10^4^ cells/well. Serum-free starvation was performed overnight, when cells reached 70%–80% confluence. Cells were then treated with serum-free medium at 312, 400, 450, 500, and 550 mOsm. After 24 h, the culture medium was removed and 100 μL basic culture medium containing 10% CCK-8 (Dojindo, Tokyo, Japan) was added; cells were incubated in the dark for up to 3 h and the absorbance (wavelength, in 450 nm) was measured by Infinite 200pro Microplate Reader. The absorbance value was recorded to determine the relative cell viability. After the background value had been subtracted, the resulting absorbance value was compared with that of the normal group. Several concentrations of CMC and α-MSH were pre-incubated for 30 min before hyperosmotic treatment. The same CCK-8 method was used to screen these concentrations.

### Scratch assay

Before cell seeding, a horizontal line and five vertical lines were drawn at 0.5-cm intervals on the back of a transparent six-well plate to allow localization of cells during image acquisition. Cells were seeded in six-well plates at a density of 5 × 10^5^ cells/well. An even line was rapidly drawn through the cell monolayer by using a 200-µl pipette tip, following overnight culture without serum at 90%–100% confluence. The suspended cells were removed by two washes with Dulbecco’s PBS. Images were immediately acquired using an optical microscope: five images per well, 10 images per group. Images were also acquired at 4, 12, and 24 h after treatment. All images were processed with ImageJ software.

### Transepithelial electrical resistance assay

HCECs were seeded in a transwell dish (Corning, USA) at a density of 3.3 × 10^4^ cells/well; each transwell was 6.5 mm in diameter, with a 0.4-μm filter diameter. Resistance was detected by a resistance tester at 2-day intervals; the medium in the upper and lower chambers was replaced during each measurement. Before resistance measurement, electrode calibration was performed. The long end of the electrode was passed through the space beside the upper chamber and pressed against the lower chamber vertically; the short end of the electrode was then inserted into the medium of the upper chamber without touching the bottom of the chamber. Stable readings were recorded and each measurement was repeated three times. Resistance values were recorded as the reading minus the background value.

### Measurement of ROS level

HCECs were seeded in 96-well plates at a density of 1 × 10^4^ cells/well, eight wells per group. Serum-free starvation was incubated overnight when cells were at 70%–80% confluence; all procedures were performed in accordance with the instructions of the ROS Measurement Kit (Sigma).

### Measurement of caspase-1 activity

HCECs were seeded in 12-well plates at a density of 2.5 × 10^5^ cells/well (five groups, six wells per group). After hyperosmolarity treatment, cells were washed twice and 100 μL of lysis buffer were added to each well; cells were detached from the plate surface with cell scraper and incubated on ice for 10 min; all remaining procedures were performed in accordance with the instructions of the Caspase-1 Detection Kit (Abcam). Data were processed by subtracting the background value of each well and ratios of protein concentrations were calculated.

### RNA isolation and real-time polymerase chain reaction (PCR)

Cells were seeded in 12-well plates at a density of 2.5 × 10^5^ cells/well. Cells were collected and frozen in liquid nitrogen after treatment. Total RNA was extracted using a GeneJET RNA Purification Kit (Thermo Fisher Scientific, Waltham, MA, USA), in accordance with the manufacturer’s instructions. The concentration and purity of total RNA were examined using a Nanodrop 2000 (Thermo Fisher Scientific). Following digestion with DNase I, 1 μg of total RNA was reverse transcribed using a RevertAid cDNA synthesis Kit (Thermo Fisher Scientific). mRNA expression levels of *NLRP3* and *IL-1β* were detected by real-time PCR using a HT7900 Real-Time PCR System (Applied Biosystems, Foster City, CA, USA). The reaction mixture contained 3 μL cDNA template, 5 μL SYBR Green FastStart 2X Master Mix (Roche, Branford, CT, USA), and 0.3 μL gene-specific primers (Table 1). The program was composed of 2 min at 50 °C and 10 min at 95 °C, followed by 40 cycles of 15 s at 95 °C and 1 min at 60 °C. A dissociation stage was added to check the amplicon specificity. This procedure was performed in accordance with our previously published method^11^. Relative expression levels were analysed using a comparative threshold cycle (2^−∆∆Ct^) method and normalised to *GAPDH* gene expression.

**Table 1:** Primers used for real-time PCR.

Gene	Sequence
*NLRP3*	Forward: 5′-TGCCCGTCTGGGTGAGA-3’
	Reverse: 5′-CCGGTGCTCCTTGATGAGA-3’
*CASPASE- 1*	Forward: 5′-TCACTGCTTCGGACATGACTACA -3’
	Reverse: 5′-GGAACGTGCTGTCAGAGGTCTT-3’
	Reverse: 5′-CCTTGCAGGTCCAGTTCCA-3’
*IL-1β*	Forward: 5′-CACGATGCACCTGTACGATCA-3’
	Reverse: 5′-AGACATCACCAAGCTTTTTTGCT-3’
*β-Actin*	Forward: 5′-CCCAGCCATGTACGTTGCTA-3’
	Reverse: 5′-TCACCGGAGTCCATCACGAT-3’

### Western blot

Cells were seeded in six-well plates at a density of 5 × 10^5^ cells/well. Cells were washed twice with cold PBS, then detached from the plate surface with a cell scraper. Following mixture with 200 μL RIPA (containing 1% protease inhibitor), cells were incubated on ice for 20 min. Supernatants were collected after high-speed centrifugation (12000 rpm, 15 min); they were then snap-frozen in liquid nitrogen and stored at − 80 °C. Protein concentrations were determined by a Bicinchoninic Acid Protein Assay Kit (CWBIO, Beijing, China). Western blots were conducted as previously described^11^. In brief, equal amounts of protein from each sample were resolved in loading buffer and boiled for 5 min. The proteins (30 μg/lane) were separated on a sodium dodecyl sulfate polyacrylamide gel, then electrotransferred to polyvinylidene difluoride membranes. The blots were washed twice with Tris-buffered saline plus Tween (TBST; 20 mM Tris [pH 7.4], 0.9% NaCl, and 0.1% Tween-20), blocked with 5% bovine serum albumin in TBST for 2 h, and incubated with primary antibodies: rabbit anti-NLRP3 (1 μg/ml) and rabbit anti-caspase-1 (3 μg/ml) (Novus Biologicals, USA) at 4 °C overnight. Following three washes with TBST, the membranes were incubated with the corresponding horseradish peroxidase-conjugated secondary antibodies (1:5000, Abcam) at room temperature for 1.5 h. Signals were detected with chemiluminescence reagents (Millipore, USA) and imaged by a Multispectral Imaging System (Biospectrum AC Chemi HR 410, UVP, CA, USA). An anti-GAPDH antibody (1:5000, CST, MA, USA) was used as an internal standard. Optical densities of target proteins were quantified by ImageJ and normalised to the density of GAPDH.

### ELISA assay

IL-1β expression levels in cell culture were determined using an ELISA kit (Enter life, UK). Cells were seeded in six-well plates at a density of 5 × 10^5^ cells/well. Cell stimulation (normal, hyperosmolarity, CMC, α-MSH, CMC + α-MSH) was performed as above. After 24 h, cell supernatants were collected for ELISA analysis. ELISA procedures were performed in accordance with the manufacturer’s instructions.

### Statistical analysis

All data were analysed by GraphPad Prism6 (version 6.00 for Mac OS X). The data are presented as means ± standard deviations. One-way analysis of variance was used for comparisons among groups when data exhibited normal distributions; the Tukey test was used for post hoc analysis. The Kruskal–Wallis test was used for comparisons when data did not exhibit normal distributions; Dunnett’s test was used for post hoc analysis. To compare differences among groups at different time points, two-way analysis of variance was used. *P* < 0.05 was considered to indicate statistical significance.

## Supplementary Information

Supplementary Information.

## Data Availability

The data are available from the corresponding author on reasonable request.
