# Supplementary material for: A combination of CMC and α-MSH inhibited ROS activated NLRP3 inflammasome in hyperosmolarity stressed HCECs and scopolamine-induced dry eye rats
Source: Sci Rep. 2021 Jan 13;11:1184. doi: 10.1038/s41598-020-80849-2 (PMC7807058; doi:10.1038/s41598-020-80849-2)

**A Combination of CMC and α-MSH Inhibited ROS Activated NLRP3 Inflammasome in Hyperosmolarity Stressed HCECs and Scopolamine-Induced Dry Eye Rats**

Ying Lv*^1^, Chenchen Chu*^1^, Ke Liu^1^, Yusha Ru^1^, Yan Zhang^1^, Xiaoxiao Lu^1^, Yichen Gao^1^, Caijie Zhang^1^, Shaozhen Zhao^1^

**Supplemental Figure legends:**

**Supplemental Figure1.** Tight junction formation by HCECs in normal culture. Three samples were taken from different cell sites.

**Supplemental Table1.** Table shows the raw data of Supplemental Figure1.

|  | Normal1 | Normal2 | Normal3 |
| --- | --- | --- | --- |
| D3 | 48 | 46 | 54 |
| D5 | 227 | 225 | 235 |
| D7 | 204.5 | 246 | 363 |
| D9 | 92 | 128 | 145 |

**Supplemental Figure2. MCC950 concentration screening.** mRNA levels of NLRP3 among groups. Relative expression levels were analysed using the comparative threshold cycle (2^−∆∆Ct^) method and normalised to GAPDH gene expression. Data are shown as mean±SEM. *, comparison of dry eye group and normal group; ^, comparison of MCC950 treatment group and hypertonic group; ns, not significant. ^ *p*<0.05; ***p*<0.01.


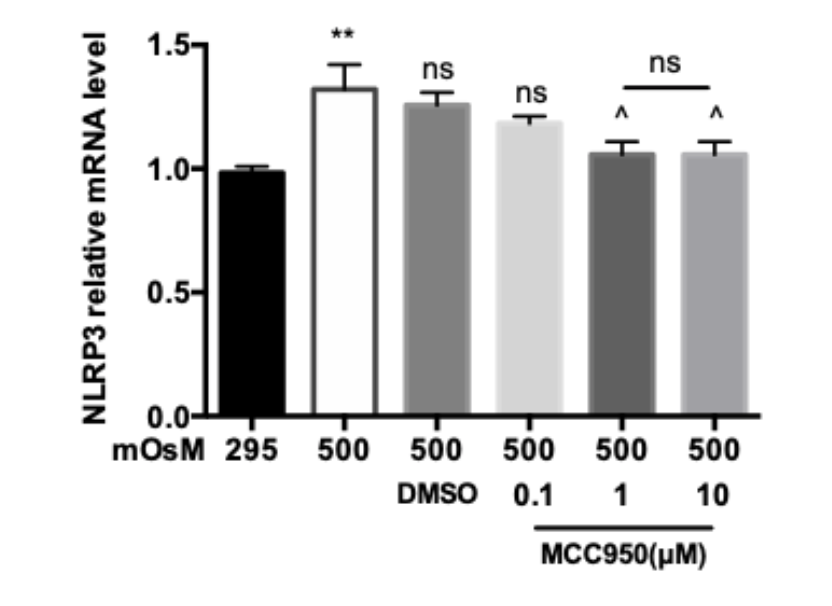


**Supplemental Figure3. Pro-caspase- 1 expression level in mRNA and protein was decreased by caspase-1 siRNA.**

**before**

**
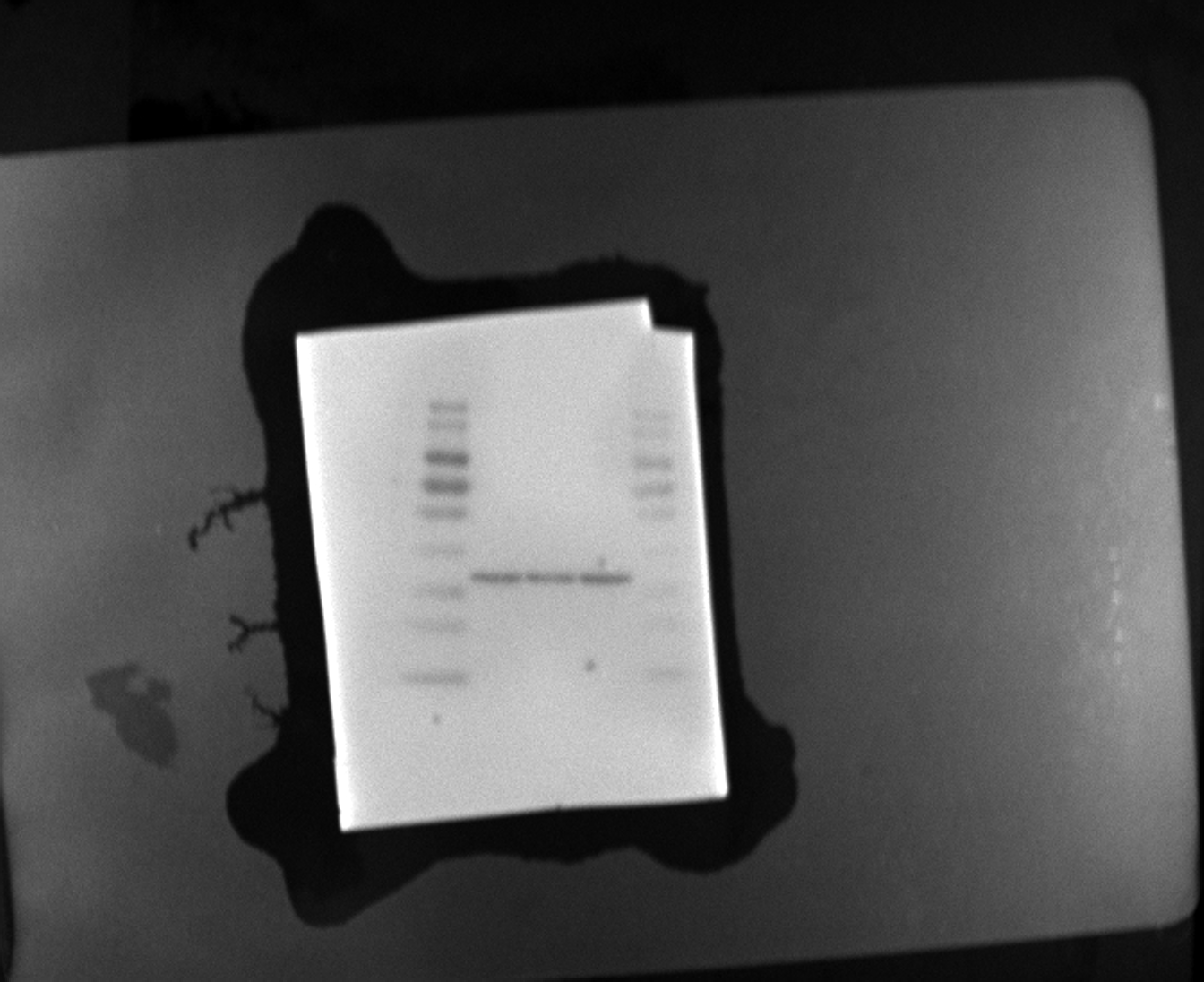
**

gapdh

caspase-1

**after**

**Supplemental Figure4. NLRP3 expression level in mRNA and protein was decreased by NLRP3 RNA.**

before

**
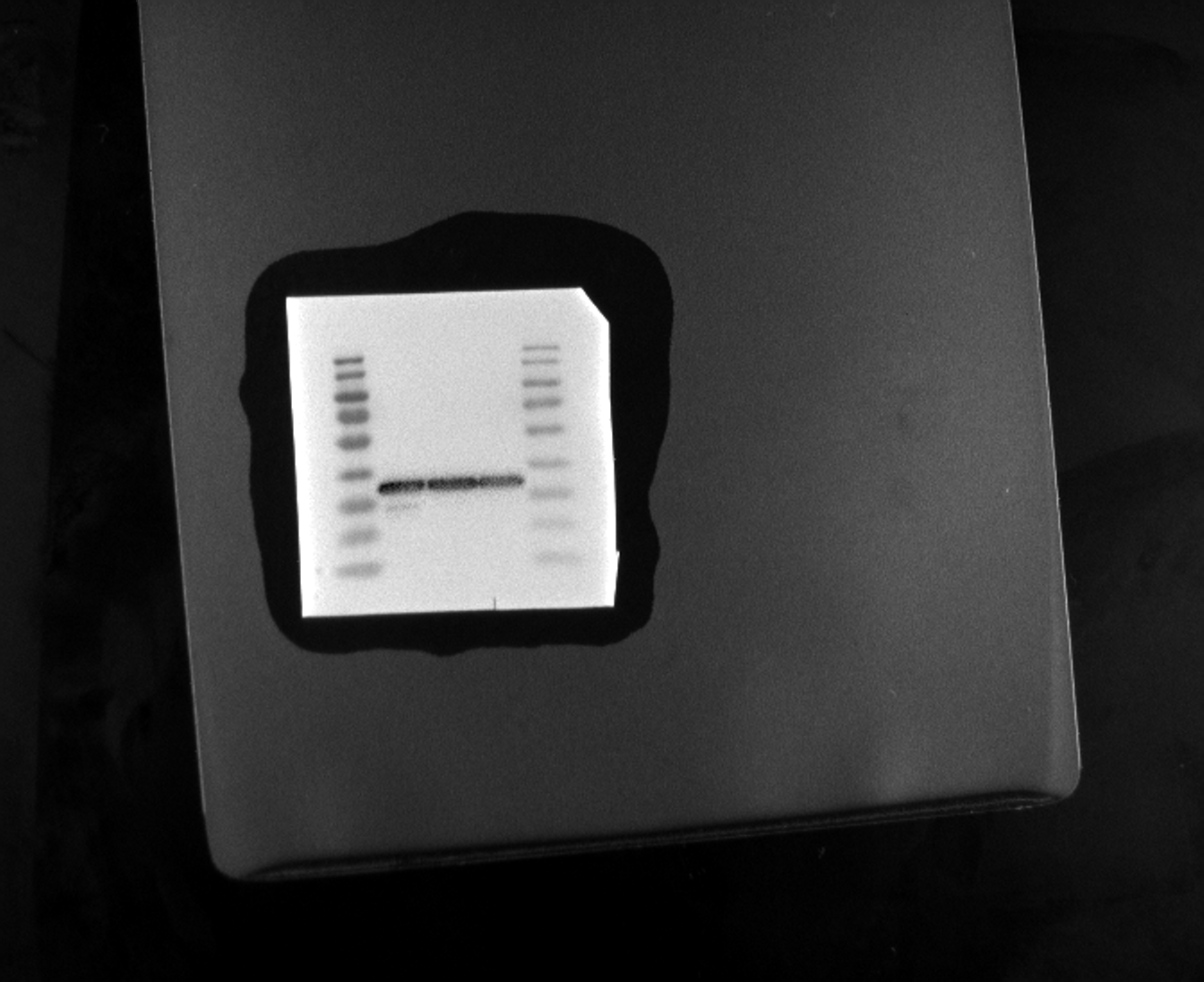
**

NLRP3 gapdh

**after**

**Supplemental Table2. sequence and target of siRNA.**

| Gene | Sequence |
| --- | --- |
| *NLRP3 siRNA* | Interference target: GGTGTGGAATTAGACAAC |
|  | Forward: 5’-GGUGUUGGAAUUAGACAACTT -3’ |
|  | Reverse: 5’-GUUGUCUAAUUCCAACACCTT -3’ |
| *CASPASE- 1*  *siRNA* | Interference target: CCTGTGATGTGGAGGAAAT |
|  | Forward: 5’-CCUGUGAUGUGGAGGAAAUTT -3’ |
|  | Reverse: 5’-AUUUCCUCCACAUCACAGGTT -3’ |

Figure 7A. original figure

NLRP3:

before:


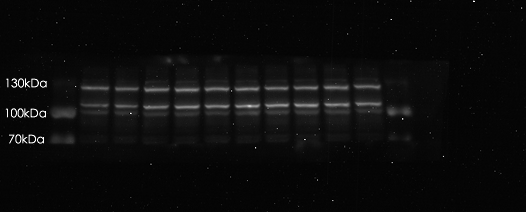


after:


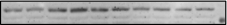


GAPDH

Before


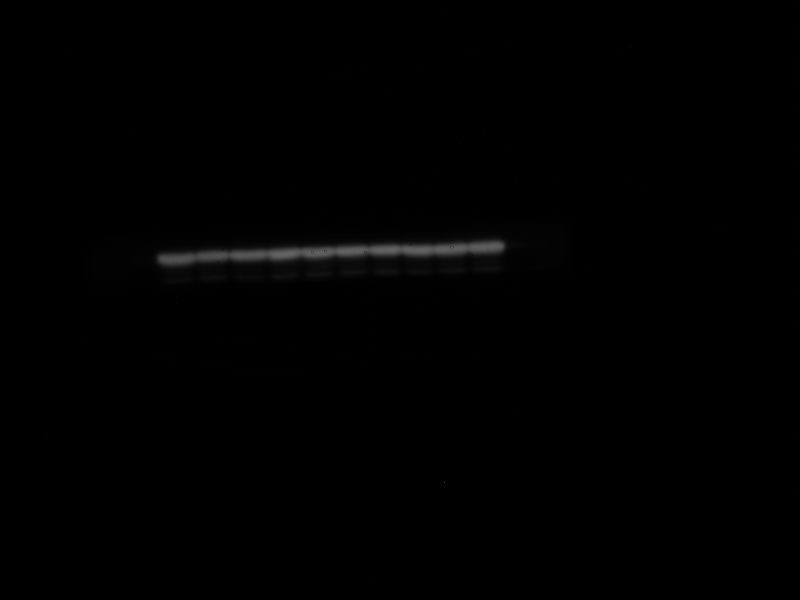


after


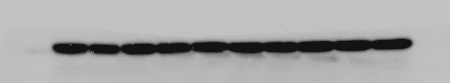


pro-CASPASE-1

before:


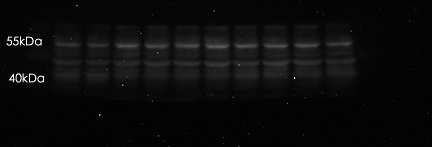


after:


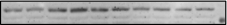


cleaved-CASPASE-1

before:


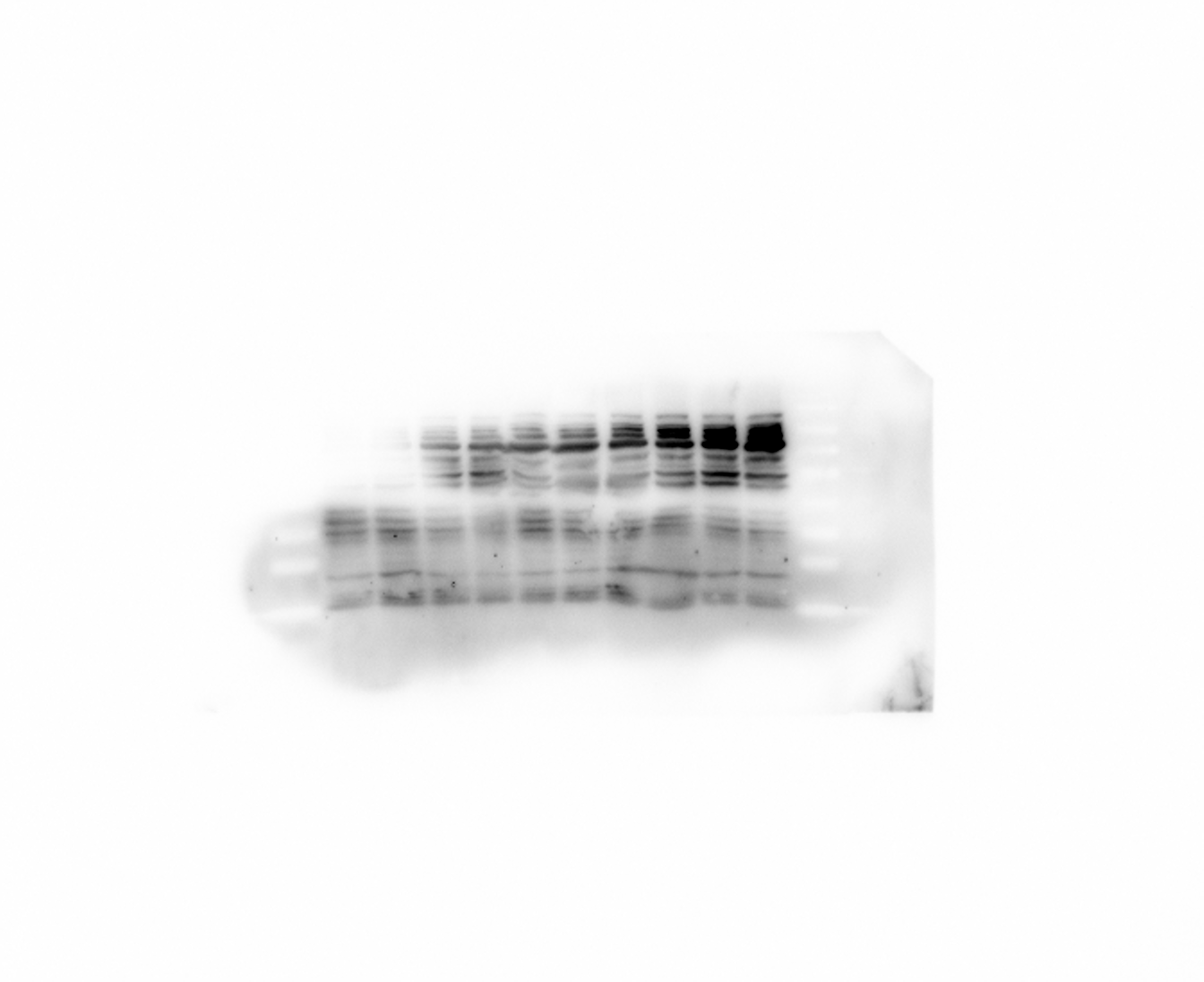

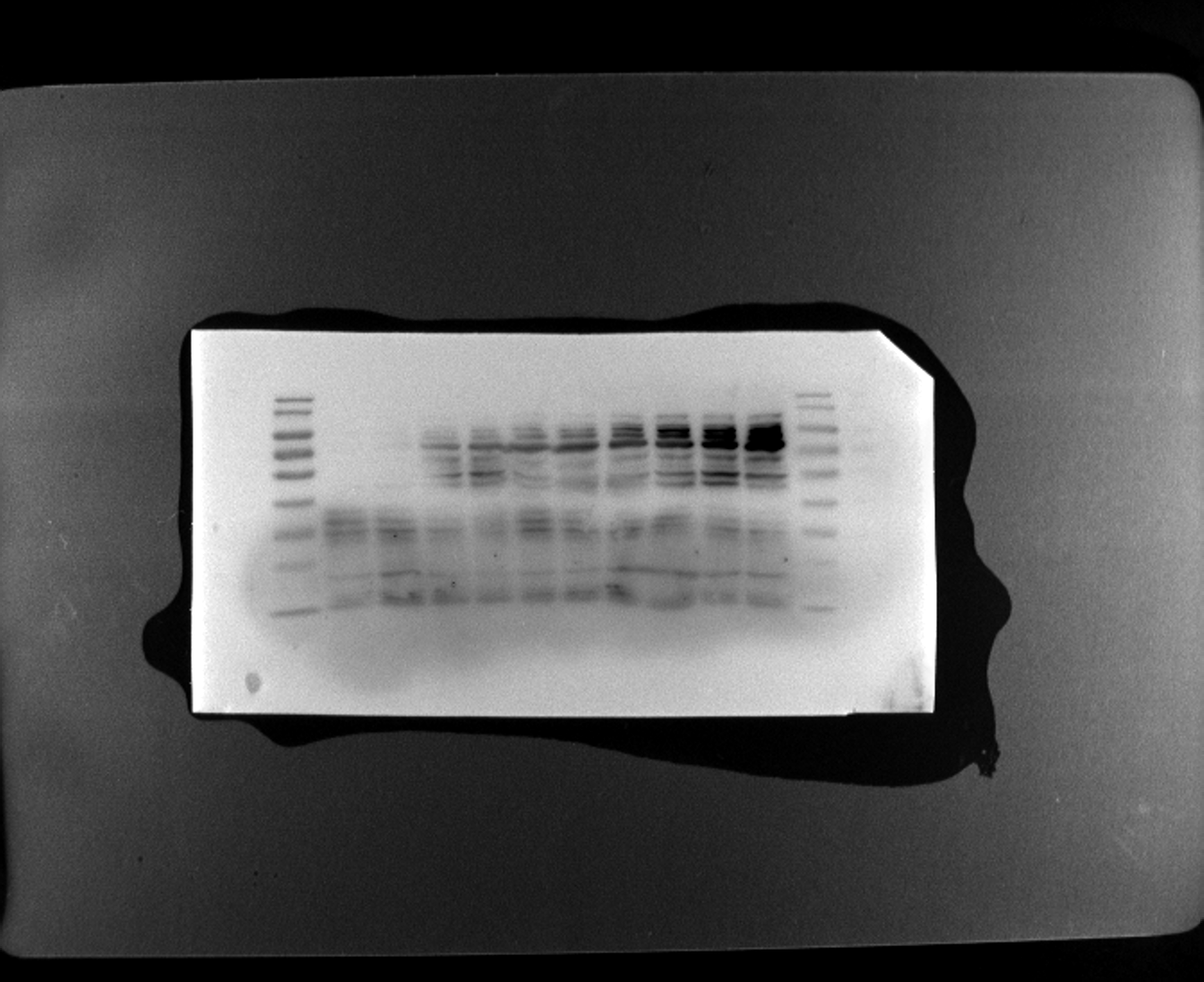


after:


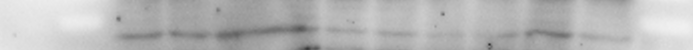


figure 8C: original figure

NLRP3 before:


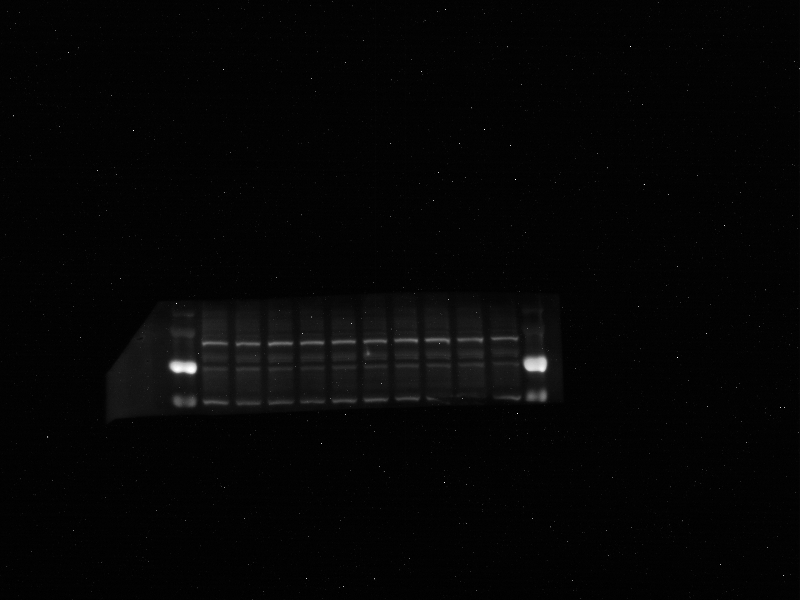


after:


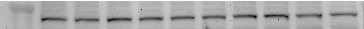


GAPDH

before


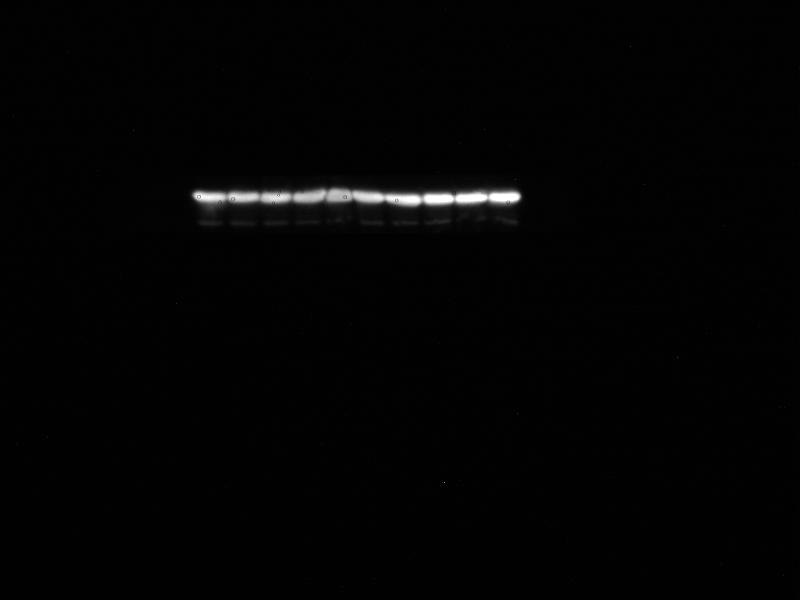


after


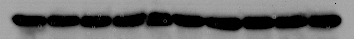

Supplement: Supplementary file 1 — Supplementary Information. [file 41598_2020_80849_MOESM1_ESM.docx]
